# Caregiving Profiles: Demographic And Economic Social Determinants Of Health

**DOI:** 10.1093/geroni/igaf122.3316

**Published:** 2025-12-31

**Authors:** S Alison Bolling, Julie Hicks-Patrick

**Affiliations:** West Virginia University, Morgantown, West Virginia, United States; West Virginia University, Morgantown, West Virginia, United States

## Abstract

In 2021-22, approximately 37.1 million Americans provided care to family or friends (U.S. Bureau of Labor, 2024). Caregiver strain may differ across age, gender, race (Young et al., 2019). Given the effects of Social Determinants of Health, such as financial stability, health care, and food resources (Duran & Perez-Stable, 2019), some adults may experience additional threats to health and quality of life before entering the caregiver role. Little is known about those who anticipate becoming a caregiver. Knowing key information about anticipated caregivers would allow better programs and policies for future caregivers. The current study includes 97,914 participants (Mean age = 55.56, 45.8% female, 81.6% white non-Hispanic) who completed the 2022 BRFSS. Approximately 20.1% were current caregivers, 10.47% anticipated becoming a caregiver in the next two years, 5.3% were unsure, and 64.11% did not anticipate becoming a caregiver. A multinomial logistic regression examined the contributions of key demographic characteristics and economic social determinants of health (eSDOH) to predicting group membership, χ²(60675) = 1832.60, p < .001. Compared to current caregivers, anticipated caregivers were more likely to be: younger (OR = .99), male (OR = 1.2), and unemployed (OR = .81). Challenges related to eSDOH were higher for current than for anticipated (OR = .89), unsure (OR = .76), and those not anticipating becoming a caregiver (OR = .73). Additional post hoc analyses compared anticipated caregivers to unsure and not anticipating. More resources should be available for those prior to becoming a caregiver to alleviate future caregiver strain.

